# What predicts the alleviation of Covid-related future anxiety in schoolchildren 6 to 9 months into the pandemic?

**DOI:** 10.3389/fpsyg.2023.1230301

**Published:** 2023-09-20

**Authors:** Katharina Voltmer, Maria von Salisch

**Affiliations:** Institute for Sustainability Education and Psychology, Leuphana University Lüneburg, Lüneburg, Germany

**Keywords:** Covid-19, future anxiety, children, classroom climate, resilience

## Abstract

**Introduction:**

Although the first COVID-19-related lockdown in the Spring of 2020 has contributed to an increase in mental health problems in many children worldwide, less is known about the longer-term effects of the pandemic on their (future) anxiety. This article examines resilience factors against children’s Covid-relatedfut ure anxiety (CRFA).

**Methods:**

*N* = 140 children (48,6% female) in 3rd and 4th grade classrooms in Northern Germany were asked to self-report about their CRFA, their anxiety, and the social climate in their classrooms in September (T1) and December 2020 (T2).

**Results:**

Results indicate that 18.6% of the children experienced CRFA “often” in at least one item of the CRFA scale at T1. CRFA was more pronounced in girls and in children from immigrant families. Changes in children’s CRFA between T1 and T2 were predicted by changes in their anxiety and changes in classroom climate. Children in classrooms with increasing levels of peer support tended to have decreasing levels of CRFA, whereas their agemates’ CRFA in less supportive classrooms tended to increase over time.

**Discussion:**

These results suggest that peer and teacher social support may bolster children’s resilience against future anxiety in challenging times. Implications for teachers and schools are discussed.

## Introduction

The COVID-19 pandemic and the ensuing health and safety measures have unsettled schools with closures and an increase in distance education. These measures have disrupted routines in many families and increased feelings of social isolation in many children (Loades et al., 2020). In a systematic review, which included the results from 21 longitudinal studies with more than 96,000 young people, Kauhanen et al. (2023) established that during the first lockdown in March to June 2020, children’s and adolescents’ mental health deteriorated in many countries. In a representative study with *N* = 1,923 parents of 7- to 17-year-olds during the first lockdown in Germany, for example, more parents reported that their children suffered from borderline or abnormal mental health problems than parents in the equally representative sample before the pandemic. More youth endorsed more clinically relevant symptoms of anxiety during the first lockdown than before the pandemic (24% vs. 15%) (Ravens-Sieberer et al., 2021). In the second wave of this study during the second lockdown in Germany (December 2020 to January 2021), the rate of young people with symptoms of generalized anxiety rose to 30%. Parent ratings of borderline and abnormal emotional problems of their seven- to eleven-year-olds likewise grew from the first lockdown to the second lockdown (Ravens-Sieberer et al., 2021).

### Covid-related future anxiety

For some people, the future is a realm of unlimited possibilities; for others it is a space of uncertainty and a cause for endless worry. Future anxiety was defined by Zaleski (1996) as a “state of apprehension, uncertainty, fear, worry, and anxiety about unfavorable changes in a more distant personal future” (Zaleski, 1996, p. 165). The object of future anxiety are the events which may threaten an individual’s personal future, that is, socially relevant events such as wars or natural disasters, or personally relevant events, such as health problems or loss of loved ones. Expecting predominantly adverse changes in the future not only affects adults but also influences children’s and adolescents’ attitudes, decisions, and behaviors by lowering their expectations of positive outcomes of their actions or by avoiding thoughts about the future altogether. People with this mindset are less inclined to recognize opportunities because they are preoccupied with the risks (Zaleski, 1996). Youth with pronounced future anxiety who behave in a wait-and-see manner limit their personal and educational aspirations (Zaleski, 1996; Seiffge-Krenke et al., 2012). Longitudinal evidence suggests that adolescents’ level of future anxiety predicted their depressive symptoms at age 35 and 45 in a large representative study (Allemand et al., 2022). Based on his theoretical work, Zaleski (1996) developed the Future Anxiety Scale for adults and condensed it into the Dark Future Scale (Zaleski et al., 2019). Because of the potentially debilitating nature of future anxiety, the Dark Future Scale was adapted to primary school children (Voltmer and von Salisch, 2021, see Supplementary material).

In the present short-term longitudinal study, level, stability, and predictors of children’s Covid-related future anxiety (CRFA) in Germany were examined. The first measurement was obtained 3 months after the end of the first lockdown at the beginning of the new school year in September 2020 (T1) and the second after 3 months of opening windows every half hour, frequent testing, and social distancing in schools in December 2020 (T2). We expect a general increase in CRFA because of the rapidly rising COVID-19 infection rate which led to school closures and a second nationwide lockdown in Germany right after T2.

### Resilience against high levels of CRFA

Masten (2001) formulated ten characteristics of children and their environment that had emerged as well-established correlates of children’s competence and psychopathology. This so called “short list” of multisystem resilience factors include resources on the individual level, e.g., self-regulation, self-efficacy, agency, and optimism, and resources on the social level, e.g., sensitive caregiving, family management, and close relationships (also see Masten et al., 2021). Studies found that some of these resilience factors were also associated with lower levels of child and adolescent mental health problems during the COVID-19 pandemic: On the individual level, a longitudinal study showed that youths with greater self-efficacy showed smaller increases in mental health symptoms during the first COVID-19 related lockdown (Hussong et al., 2021). No buffering effect of child optimism against the jump in irritability, hopelessness, sadness, loss of interest in friends, fighting with other children, or having trouble sleeping was found in this study with 100 preadolescents from the US. Problem-focused engaged coping seemed to shield preadolescents against the rise in symptomatology during the first lockdown. Thus, self-efficacy and problem-focused engaged coping seemed to be resources against the rise in symptomatology in response to the social isolation of the first lockdown at the level of individual children (Hussong et al., 2021).

On the social level, social-support seems to be an effective resilience factor for children and adolescents (Seiffge-Krenke et al., 2012). For children, family support emerged as a resource during the lockdown. A diary study demonstrated that parents’ authoritative parenting of their 6- to 19-year-olds over the 21 days of the study during first lockdown contributed to children’s and adolescents’ positive affect and lowered their emotional problems during this period of strain for the whole family (while controlling for relevant covariates; Neubauer et al., 2021). Family support also seemed to moderate changes in anxiety during the pandemic. In the German representative study described above, higher levels of family support were associated with a reduction in anxiety and depressive symptoms from the first to the second lockdown (Ravens-Sieberer et al., 2021). Experiencing social support in the family thus seems to increase children’s resilience against the strain generated by the COVID-19 pandemic.

A less explored social resource may lie in supportive teacher – child or peer relationships. Primary school children in Germany spend 5 to 8 hours every school day in the company of the same classmates and teachers. In some classrooms children experience mainly supportive relationships to their classmates, whereas in others some children are ostracized by their peers because of apparent flaws. Being shunned is likely to increase children’s anxiety (Wang et al., 2020). In a large cohort study in Great Britain, having peer problems in adolescence was strongly associated with more anxiety symptoms whereas prosocial behavior was associated with fewer anxiety symptoms (Konac et al., 2021). In a recent study, students’ perception of supportive peer relationships in the classroom increased their sense of community which in turn bolstered their social well-being (Capone et al., 2018). Being part of a positive (learning) community seems to fulfill children’s basic psychological need of relatedness (Deci and Ryan, 2008). The second question therefore examines whether supportive peer relationships in the classroom serve as a resource against changes in individual children’s CRFA between T1 and T2. We expect that an increasingly supportive mean peer classroom climate mobilizes social support and builds resilience against CRFA in individual children between T1 and T2.

Sociodemographic and socioeconomic factors like age, gender, maternal education, family composition, and parental mental health were also found to be associated with young peoples’ general anxiety symptoms (Dooley et al., 2015). They are likely to affect CRFA as well. Because the COVID-19 pandemic has affected families of a lower *socio-economic status* (SES) more severely, children from these families tended to experience more anxiety than children from more affluent families (e.g., Ravens-Sieberer et al., 2021; Voltmer and von Salisch, 2023). Children from *immigrant families* (who often find themselves at the low end of the SES distribution) tended to have more total mental health problems (but did not show higher levels of emotional problems or generalized anxiety during the first lockdown; Ravens-Sieberer et al., 2021). *Gender differences* in anxiety are well known. Girls tend to report more intensive (future) anxiety than boys (e.g., Essau et al., 2002; Seiffge-Krenke et al., 2012; Kästner et al., 2022). Thus, SES, family migration, parent education, and gender will be included in all analyses.

### The current study

The present short-term longitudinal study examines the course of children’s CRFA. In summary, the aim of the present research is (1) to describe level and stability of children’s CRFA 6 and 9 months into the COVID-19 pandemic in Germany and (2) to examine social resilience factors with the expectation that mean levels of supportive peer relationships in the classroom contribute to the explanation of a decrease in children’s CRFA between T1 and T2 when background factors are included in the analyses.

## Method

### Sample

The participating classes consisted of *N* = 140 children from *N* = 9 third- and fourth- grade classrooms in Northern Germany with *n* = 68 (or 49% female participants). Children’s age ranged between 7:11 and 11:2 years at T1 with a mean of 8:11 years (SD 7.64 months). At T2 eight children dropped out of the T1 sample of *N* = 140, but five children who had not been present at T1 joined the study. Thus, sample sizes for T1 and T2 were *N* = 140 and *N* = 137, respectively. The reasons for missing measurements and drop-out were illness, moving to another city, and refusal to participate. Of the T1 sample, 94% participated in the T2 data collection.

Parents’ questionnaires were used to record the families’ migration history and their socioeconomic background in terms of the highest educational attainment within the family. In the T1 sample, *n* = 29 children (21%) had one or two parents who were born outside of Germany. Parents’ highest educational attainment could be calculated for *n* = 134 children (90%). In five families (4%) parents had dropped out of school before graduation, in 18 families (13%) at least one parent held a secondary school diploma (or comparable diplomas), in seven families (5%) at least one parent obtained the Abitur, the general qualification for admission to university in Germany. In 24 families (18%) at least one parent had undergone a vocational training (e.g., an apprenticeship), in 12 families (9%) at least one parent had finished a Technical College, and in 68 families (51%) one (or more) parents held a university degree. Overall, in *n* = 104 families (76%), at least one parent had a vocational qualification.

Between T1 and T2, *n* = 81 children from five classrooms were randomized to participate in a breath-based mindfulness program. Due to this embedded intervention, we control for treatment exposure (coded as 0 = control and 1 = treatment) in subsequent analyses although no effects on children’s future anxiety were found to distinguish the participating students from the *n* = 59 who did not participate.

### Procedure

After obtaining permission from the Ethics Review Board of our university (on May 6, 2020), T1 data were collected in September and T2 data in December 2020 just before the second COVID-19-related lockdown in Germany. Children entered their data in the tablets we provided for them in the classroom or in small group settings. Items and instructions were read aloud by a test manager in front of the class. Three additional trained undergraduate research assistants supported children with technical difficulties or insufficient computer skills. Children took part in the study only after they had brought the written consent of a parent or guardian. Children’s participation was voluntary. The survey lasted three to four teaching hours at each measurement point and was interrupted by the usual breaks between the lessons. Children who did not participate quietly occupied themselves with materials provided by the teacher. At the end of each data collection, all children received a small gift. Together with their consent form, parents completed a short questionnaire about their family situation, the language(s) spoken at home, and their educational and professional background.

### Measures

#### Covid-related future anxiety

Children’s CRFA was assessed at all measurement points with the Epidemic-Related Dark Future Scale for Children (eDFS-C; Voltmer and von Salisch, 2021). This scale was adapted from the German version of the Dark Future Scale for adults (Zaleski et al., 2019; Dadaczynski et al., 2022). When adapting the Dark Future Scale for primary school children, five items were condensed into four items and the language was adapted to children’s more concrete level of understanding. Therefore, items were turned into questions which started with “Are you afraid that…” and the COVID-19 virus was mentioned in each item (see Supplementary material). In the future, the name of another specific epidemic or pandemic can be inserted into the items. The four items were scored on a four-point scale (0 = “never,” 1 = “seldom,” 2 = “sometimes,” 3 = “often”). No item was reverse scored. Raw scores could range from 0 to 12 with higher scores indicating more CRFA. An example item is “Are you afraid that your life may get worse due to the COVID-19 virus?” In the present study, internal consistency was acceptable with *α* = 0.76 at T1 and *α* = 0.73 at T2. Kästner et al. (2022) likewise obtained an acceptable internal consistency of *α* = 0.77 in their sample of *N* = 840 8- to 18-year-olds in Germany. The eDFS-C overlaps partially but not completely with other measures obtained in this study, i.e., self-reports of anxiety (T1: SCAS- social phobia *r* = 0.46, *p* < 0.001, SCAS- generalized anxiety *r* = 0.55, *p* < 0.001; see below) and teacher reports of emotional problems on the SDQ (*r* = 0.19, *p* < 0.05). That children who had experienced the effects of the COVID-19 virus in their family tended to report higher levels of CRFA speaks for the criterion validity of the eDFS-C (Voltmer and von Salisch, 2021). For the variable *change in CRFA*, children’s T1 scores were subtracted from their T2 scores.

#### Anxiety

Anxiety was measured at T1 with the Spence Children’s Anxiety Scale (SCAS; Spence, 1998; German translation by Essau et al., 2002) which examines symptoms of specific anxiety disorders. For lack of time, only the subscales “social phobia” (six items) and “generalized anxiety disorder” (six items) of the SCAS were used. Responses were scored on a four-point scale (0 = “never,” 1 = “sometimes,” 2 = “frequently,” 3 = “always”). Scores were summed for the 12 items (with a maximum score of 36), with higher scores indicating more anxiety symptoms. In the norming study, test–retest reliability was 0.57 (social phobia) and 0.56 (generalized anxiety). Internal consistency was acceptable with *α* = 0.70 for social phobia and *α* = 0.73 for generalized anxiety (Spence, 1998). In the present study we used the combined scale of social phobia and generalized anxiety to the scale *anxiety* with an internal consistency of *α* = 0.82 at T1 and *α* = 0.80 at T2. For the variable *change in anxiety*, children’s T1 scores were subtracted from their T2 scores.

#### Classroom climate

The subscale “classroom climate” of the German questionnaire to assess the emotional and social school experiences of third and fourth grade school children (FEESS 3–4) was used at T1 and T2 to measure children’s perceptions of the social climate among the classmates. Classroom climate is defined in the manual as the “extent to which the children in the class interact in a socially appropriate and friendly manner and have a good relationship with each other” (Rauer and Schuck, 2003, p. 9, authors’ translation). The subscale includes 11 items that are scored on a Likert-type scale (0 = “not at all true,” 1 = “hardly true,” 2 = “somewhat true,” 3 = “exactly true”). Six items were reverse scored. Raw scores could thus range from 0 to 33 with higher scores indicating a more supportive classroom climate. An example item is “We make fun of some children” (reverse scored). Internal consistency was acceptable with *α* = 0.72 at T1 and *α* = 0.83 at T2 in the present study. Children’s scores within a classroom were summed and then divided by the number of participating children in the classroom at T1 and T2. For the variable *change in mean classroom climate*, children’s mean classroom T1 scores were subtracted from their mean classroom T2 scores (*α* = 0.67).

## Results

All statistical analyses were conducted with the software IBM SPSS Statistics Version 28. Before the main analyses data were screened for outliers and multicollinearity. Bivariate Pearson correlations and descriptive statistics for the variables are displayed in Table 1.

**Table 1:** Intercorrelations between the variables.

	1	2	3	4	5	6	7
1. Gender (0 = boy, 1 = girl)							
2. Parent education (0 = school,1 = vocational)	−0.15						
3. Family migration (0 = no, 1 = yes)	−0.03	−0.51^***^					
4. Treatment (0 = CG, 1 = IG)	−0.13	0.12	−0.13				
5. Change in CRFA T1–T2	0.02	0.02	0.05	−0.03			
6. Change in anxiety T1–T2	−0.10	0.04	0.07	−0.03	0.34^***^		
7. Change in mean classroom climate T1–T2	0.01	−0.07	0.18^*^	0.45^***^	−0.154	−0.050	
Mean	–	–	–	–	−0.05	0.97	0.91
SD	–	–	–	–	3.30	5.06	2.61
Range	–	–	–	–	19.00	24.00	8.23
Skew	–	–	–	–	0.42	−0.18	−0.20
Kurtosis	–	–	–	–	0.42	−0.32	−0.92

* *p* < 0.05; ** *p* < 0.01; *** *p* < 0.001; *N* = 128–140.

Immigrant parents were more likely to have completed fewer years in formal education whereas German-born parents were more likely to have completed a professional education (apprenticeship or university). Children from immigrant families were more likely to sit in classrooms with an increasingly supportive classroom climate. Intervention classrooms were more likely to maintain a supportive classroom climate than control classrooms. Changes in anxiety and CRFA were positively related: the more anxiety increased between T1 and T2, the more CRFA increased. Treatment correlated with change in mean classroom climate but not with changes in CRFA or any other variable.

### Level and stability of CRFA

Frequent CRFA was experienced by a minority of children which decreased over time: at T1, 18% of the children endorsed “often” in at least one of the four items of the CRFA scale, at T2 it was 14%. Thus, CRFA was somewhat stable with *r*(132) = 0.48, *p* < 0.001 between T1 and T2. When looking more closely at the changes between T1 and T2, 60% of the children remained at the same level of CRFA (+/− 2 points), while the other 40% varied in their CRFA, with large interindividual differences. As presented in Table 1, the CRFA difference score had a mean of −0.05 and a standard deviation of 3.30. Means for the level of CRFA at T1 and T2 grouped by gender and family migration are presented in Table 2. Girls reported more CRFA than boys, both at T1 and T2. Children from immigrant families reported more CRFA at T1 and T2 than native-born children (see Table 2). Children’s CRFA was not related to age (T1: *r*(135) = 0.11, *p* = 0.199., T2: *r*(127) = 0.07, *p* = 0.413). Changes in children’s CRFA over time were also not related to age (*r*(127) = −0.02, *p* = 0.856) (Table 2).

**Table 2:** Means and standard deviations of CRFA at T1 and T2 by gender and family migration.

	CRFA T1 M (SD)	*t*	*p*	CRFA T2 M (SD)	*t*	*p*
Gender						
Girl	5.35 (3.54)	−2.31	0.022	5.30 (3.23)	−2.92	0.004
Boy	4.05 (3.09)			3.74 (2.89)		
Family migration						
Without	4.36 (3.26)	−2.26	0.026	4.12 (2.99)	−2.91	0.004
With	5.93 (3.50)			6.12 (3.43)		

Notes: Covid related future anxiety. *N* = 126–138.

### Predictors of change in CRFA

In the first step of a hierarchical linear regression model (Table 3) none of the control variables treatment (intervention group vs. active control group), gender, family migration, and parents’ education was a significant predictor of change in CRFA between T1 and T2. However, the change in anxiety between T1 and T2 which was included in the second step of the hierarchical regression was a significant predictor. Additionally, change in the mean classroom climate between T1 and T2 significantly predicted change in CRFA in the third step. The negative beta indicates that the difference in CRFA between T2 and T1 became smaller when the difference in mean class climate between T2 and T1 grew larger.

**Table 3:** Prediction of change in CRFA between T1 and T2.

	Beta	Std. Error	*β*	*t*	*p*
Dependent variable: Dif. CRFA					
Step 1 (*R*^2^ = 0.01)					
Group (0 = CG, 1 = IG)	−0.227	0.621	−0.034	−0.366	
Gender (0 = boy, 1 = girl)	0.161	0.616	0.025	0.262	
Family migration (0 = no, 1 = yes)	0.673	0.889	0.083	0.757	
Parent education (0 = school,1 = vocational)	0.580	0.863	0.075	0.672	
Step 2 (Δ*R*^2^ = 0.13, *p* < 0.001)					
Dif. anxiety	0.238	0.058	0.366	4.138	***
Step 3 (Δ*R*^2^ = 0.04, *p* = 0.024)					
Dif. mean classroom climate	−0.304	0.133	−0.235	−2.291	*

* *p* < 0.05; ** *p* < 0.01; *** *p* < 0.001; *N* = 271; *R*^2^ = 0.18.

## Discussion

Although children’s time horizon is more limited than adolescents’, for whom stress about “tomorrow and the day after tomorrow” are typical (Seiffge-Krenke et al., 2012), a substantial minority of children already harbored anxiety about the COVID-19 pandemic’s ability to limit their chances in life. In September 2020, that is after a 2.5-months lockdown and 6 months into the pandemic in Germany, 18.6% of the third- and fourth graders (among them more girls than boys) endorsed at least one item indicating that they were “often” afraid that their own or their family’s life will take a turn for the worse or that they will be unable to realize their potential because of the pandemic. Not surprisingly, anxious children reported higher levels of CRFA than their less anxious classmates. Children’s CRFA was quite stable and decreased only slightly towards T2 3 months later despite rising rates of Covid infections with no vaccinations in sight. Chronic or excessive anxiety may leave traces in the developing brain (e.g., Fremont et al., 2005; Raufelder et al., 2021) and limit children’s personal, educational, and vocational aspirations.

Feeling anxious about a future, which has been made more uncertain by the advent of the (at that point) uncontrollable COVID-19 pandemic, demonstrates children’s growing awareness of the perils that may impinge on their personal futures (Seiffge-Krenke et al., 2012; Kästner et al., 2022). That girls reported more pronounced CRFA than boys at T1 and T2 corroborates the gender differences found in many other studies on anxiety because of self-presentation (e.g., Essau et al., 2002; Kästner et al., 2022). Possibly girls tend to feel more comfortable expressing anxiety than most boys do. Studies from various countries agree that children from families with less parental education tended to experience more anxiety than children from more affluent families during the first lockdown (e.g., Ravens-Sieberer et al., 2021). Children from immigrant families (whose parents have often had less of an education) tended to have more total mental health problems during the first lockdown (Ravens-Sieberer et al., 2021), because of their families’ increased risk of infection, because their families tended to live in close quarters, which promoted infections, or because parents often held low-paying jobs with much exposure to the virus, such as nursing or sales. Other reasons may have to do with parents’ level of stress, e.g., because they had to work short hours, lost their jobs altogether or because they had to educate their children in distance learning (in addition to their own job) although they themselves may not have had sufficient education for this task (OECD, 2020).

Having supportive relationships with peers seems to buffer young people against the effects of the pandemic. Social support in the sense of children’s knowledge that they are respected and cared for, and that they have a network of people concerned with their welfare (Davidson and Demaray, 2007) is known to build resilience against stress and anxiety (Seiffge-Krenke et al., 2012; Helmreich et al., 2017). The present study was conducted while children attended school every day. Results suggest that perceiving peer relationships in the classroom to be increasingly supportive contributed to a decrease of children’s CRFA during challenging times. Having classmates who take ever more care to include others in online and offline activities and who gave them instrumental and emotional support, seemed to create a sense of belonging (Deci and Ryan, 2008) which alleviated children’s anxieties about a restrictive future and the pending social isolation during the second lockdown. That children’s increasing CRFA (and anxiety) was lightened by a positive change of the social climate within the classroom resonates with evidence from a representative sample, that anxious and withdrawn children tended to react more strongly to elements of their classroom environment than their agemates who were less withdrawn (Hughes and Coplan, 2018).

That a supportive classroom climate bolstered students’ resilience amends results on teachers’ social support during the pandemic. Perceiving their teachers to be supportive lessened the effects of the first lockdown on young adolescents’ mental health (Wright and Wachs, 2022). Many primary school teachers experienced compassion fatigue (Yang et al., 2021) during the first lockdown, and the majority continued to be moderately stressed when they returned to teaching in the fall (Pressley et al., 2021). It may have been difficult for these teachers to provide the support needed by their students in order to guard them against the effects of the pandemic on their mental health. Since positive peer relationships seem to increase children’s resilience against CRFA, teachers should strive to build and maintain supportive relationships between the students in their classrooms. Perceptions of control (Pavarini et al., 2020), self-efficacy, and problem-focused engaged coping (Hussong et al., 2021) are additional factors which tended to increase children’s sense of agency in relation to the pandemic, and counteracted their feelings of future anxiety. Teachers who used problem-focused coping or job crafting (Ciuhan et al., 2022) and schools and school districts who supported social–emotional learning (Zieher et al., 2021) facilitated this process.

Contrary to expectations, only a slight decrease in CRFA between T1 and T2 was found. This may have to do with the fact that data were collected while children were in school every day. A recent systematic review indicated that social isolation and feelings of loneliness furthered the risk of internalizing symptoms in youth, especially when the isolation lasted longer (Loades et al., 2020). Children’s increasing anxiety in the German representative study may be partially explained by their social isolation during the periods of data collection which coincided with lockdowns and school closures (Ravens-Sieberer et al., 2021). When comparing the few children who attended school in an emergency program to those who were in school infrequently, parents noted that much reduced social contacts were associated with increasing mental health problems overall, more pronounced emotional problems, as well as increasing symptoms of anxiety, and depression (Ravens-Sieberer et al., 2021).

The mindfulness treatment was associated with a positive change in classroom climate. Therefore it cannot be excluded that the treatment had an indirect effect on change in CRFA via change in classroom climate. However, in the regression analysis, treatment was not a significant predictor of changes in CRFA. Nevertheless, the results may provide support for the idea that the efficacy of mindfulness interventions in schools also unfolds via changes in classroom climate. This needs to be empirically tested in future studies.

Strengths of the current study include the longitudinal examination of children’s self-reports of their CRFA, anxiety, and classroom climate 6 and 9 months into the pandemic which do not underestimate their CRFA, as reports by adults are likely to do (Lagatutta et al., 2012). Conducting the study in classrooms reduces children’s loneliness and the selection effects which can be observed in the volunteer samples of many online studies. Limitations include the study of a moderately sized sample from one region in Germany. Results need to be replicated in larger and more representative samples without an intervention. Self-reports of CRFA and anxiety may be influenced by gender stereotypes and other concerns of self-presentation. Future studies should investigate supportive peer relationships with more measures and in concert with further moderators of children’s CRFA, such as their agency, their self-efficacy (Hussong et al., 2021), their teachers’ coping and well-being, as well as family and community resiliency.

Children are a vulnerable group. This study has shown that a sizeable minority of children suffer from CRFA, which is casting a shadow onto their educational and personal futures. Social support in the classroom seems to serve as a protective factor against CRFA. Schools should therefore strive to build a supportive climate in every classroom. Peer support could also relieve teachers in their task of providing social support to counteract children’s CRFA. Future research should examine which children maintain high levels of CRFA despite the abatement of the pandemic.

## Data availability statement

The raw data supporting the conclusions of this article will be made available by the authors, without undue reservation.

## Ethics statement

The studies involving humans were approved by Ethics Committee of Leuphana University Lueneburg. The studies were conducted in accordance with the local legislation and institutional requirements. Written informed consent for participation in this study was provided by the participants’ legal guardians/next of kin.

## Author contributions

MS: conceptualization and design, supervision, original draft preparation, and reviewing and editing. KV: conceptualization and design, material preparation, data collection, data curation and data analysis, original draft preparation, and reviewing and editing. All authors contributed to the article and approved the submitted version.
