# Supplementary material for: What predicts the alleviation of Covid-related future anxiety in schoolchildren 6 to 9 months into the pandemic?
Source: Front Psychol. 2023 Sep 20;14:1230301. doi: 10.3389/fpsyg.2023.1230301 (PMC10548825; doi:10.3389/fpsyg.2023.1230301)
Supplement: Supplementary file 1 [file Table_1.docx]

**Open supplemental material**

**Dark Future Scale Short Form for Adults (Zalewski et al., 2019) adapted to the Epidemic-Related Dark Future Scale for Children**

|  | Epidemic-Related Dark Future Scale for Children (Voltmer & von Salisch, 2021) | Dark Future Scale Short Form (Zalewski et al., 2019) |
| --- | --- | --- |
| 1. | Are you afraid, that the [Covid-19 virus] may stay on for a long time? | I am afraid that the problems which trouble me now will continue for a long time |
| 2. | ---- | I am terrified by the thought that I might  sometimes face life’s crises or difficulties |
| 3. | Are you afraid that your life may get worse due to the [Covid-19 virus]? | I am afraid that in the future my life will  change for the worse |
| 4. | Are you afraid, that your family will soon be able to afford less because of the [Covid-19 virus]? | I am afraid that changes in the economic and political situation will threaten my future |
| 5. | Are you afraid that the [Covid-19 virus] will prevent you from doing your hobbies, graduating from school, or doing your dream job in the future? | I am disturbed by the thought that in the future I won’t be able to realize my goals |
